# Quantifying the efficiency of photoprotection

**DOI:** 10.1098/rstb.2016.0393

**Published:** 2017-08-14

**Authors:** Alexander V. Ruban

**Affiliations:** School of Biological and Chemical Sciences, Queen Mary, University of London, Mile End Road, London E1 4NS, UK

**Keywords:** chlorophyll fluorescence, protective non-photochemical quenching, photoinhibition, light tolerance, crops

## Abstract

A novel emerging technology for the assessment of the photoprotective ‘power’ of non-photochemical fluorescence quenching (NPQ) has been reviewed and its insightful outcomes are explained using several examples. The principles of the method are described in detail as well as the work undertaken for its justification. This pulse amplitude modulated chlorophyll fluorescence approach has been applied for the past 5 years to quantify the photoprotective effectiveness of the NPQ and the light tolerance in *Arabidopsis* plants grown under various light conditions, during ontogenetic development as well as in a range of mutants impaired in carotenoid and protein biosynthesis. The future applications of this approach for the assessment of crop plant light tolerance are outlined. The perspective of obtaining detailed information about how the extent of photoinhibition and photoprotection can affect plant development, growth and productivity is highlighted, including the potential for us to predict the influence of environmental elements on plant performance and yield of crops. The novel methodology can be used to build up comprehensive light tolerance databases for various current and emerging varieties of crops that are grown outdoors as well as in artificial light environments, in order to optimize for the best environmental conditions that enable high crop productivity.

This article is part of the themed issue ‘Enhancing photosynthesis in crop plants: targets for improvement’.

## 1. Introduction

The photosynthetic organisms of our planet first evolved in aquatic environments where light intensity is normally very low. Therefore, in the course of evolution, the microscopic photosynthetic bacteria acquired light harvesting systems, or antennae, built of various proteins carrying and coordinating many interconnected pigments capable of efficiently absorbing and delivering photon energy to the photosynthetic apparatus [1,2]. This led to an increase in photosynthetic productivity by about two orders of magnitude. Eventually, evolution allowed some photosynthetic organisms (plants) to emerge onto land. There they encountered a new challenge arising from rapid and large fluctuations in light intensity. High light exposure causes frequent saturation of the photosynthetic membrane with energy that cannot be used for photosynthesis. This excess energy potentially causes damage to the photosynthetic reaction centres, particularly of photosystem II (PSII), leading to the sustained decline of its efficiency (photoinhibition), undermining plant well-being and impacting their diversity in the natural environment and the productivity of crops [3–5].

**Figure 1.**
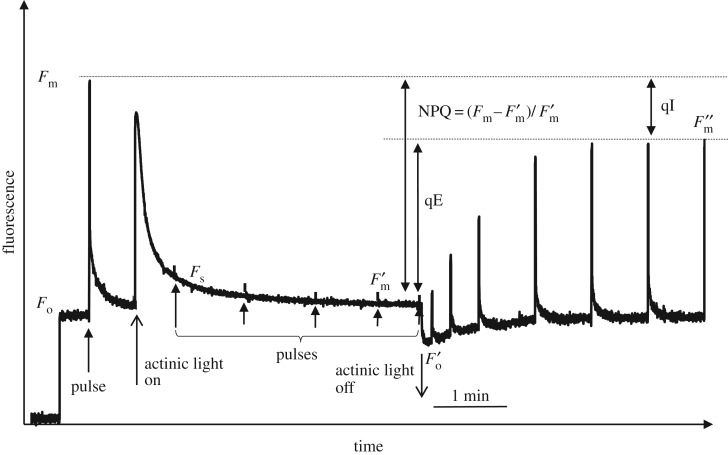
Typical PAM fluorescence measurement of an *Arabidopsis* leaf showing induction and relaxation of NPQ. *F*_m_ and *F*_o_ are the maximum and minimum fluorescence levels in the dark before actinic light illumination (1000 µmol m^−2^ s^−1^, on/off indicated by open arrows). *F*_s_ and 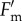 are the steady-state fluorescence and maximum fluorescence levels during actinic light illumination, respectively. 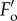 is the minimum fluorescence level after actinic light is switched off. 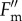 is the maximum fluorescence level following the recovery of the rapidly reversible components of NPQ. Pulses of light (indicated by vertical arrows, 10 000 µmol m^−2^ s^−1^, normally of 0.5–1.0 s duration) are applied to close all RCIIs and estimate *F*_m_ and 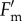. qE and qI are the quickly and slowly reversible components of NPQ, respectively. Plants were grown in plant growth chambers (Percival) with a 10 h photoperiod at 200 µmol m^−2^ s^−1^ light at 20°C. Measurements were performed on eight-week-old plants.

Non-photochemical chlorophyll fluorescence quenching (NPQ) is a phenomenon that reflects a process of prompt absorbed light energy dissipation into heat, which takes place during high light exposure in the photosynthetic membrane [6]. NPQ is broadly considered to be a major factor in the rapid regulation of light harvesting in order to protect the PSII reaction centres (RCII) against photodamage that leads to photoinhibition of photosynthesis [7]. While there is a great deal of knowledge about the elements that trigger, tune and actually cause the quenching [6,7], little is known about its protective efficiency or the critical light intensity that is ‘safe’ for the photosynthetic organism to live in, given a certain level of NPQ. While some *in vitro* studies have questioned the significance of NPQ in the protection of PSII against photodamage [8], it is commonly accepted that qE, the major, rapidly reversible NPQ component, reflects a key molecular protective process in the photosynthetic membrane of higher plants and algae, which enables rapid adjustment of light harvesting efficiency to incidental light intensity [6]. Figure 1 and §2 provide an explanation of the approach and the terminology used in so-called quenching analysis [9]. However, the nature of the remaining slowly reversible component of NPQ (defined as qI, figure 1) is highly heterogeneous, and it is believed that zeaxanthin, trapped protons, aggregated light harvesting complex II (LHCII) and photodamage to the RCII itself are the contributors to this component [10–14]. The same uncertainty is, therefore, related to the use of the PSII quantum yield, measured in the dark after relaxation of qE, as an indicator of photodamage [6]. It seems that the temporal criterion for distinguishing photoprotective from photodamaging components of NPQ, which both affect PSII quantum yield, is arbitrary and therefore ambiguous. Hence, other independent approaches are needed to verify the amount of protective NPQ as well as to distinguish between the photodamage to RCIIs and sustained downregulation by protective slowly relaxing components of NPQ. Indeed, even if NPQ is an effective adaptation to excessive light, the common occurrence of photoinhibition in nature shows that it may be limited in its protective capacity under some conditions. This means that the role of NPQ in determining plant productivity remains theoretical and un-quantified.

Assessment of photoinhibition includes the use of oxygen evolution or photosystem II reaction centre protein D1 degradation techniques as well as the previously mentioned dark-adapted PSII quantum yield (*F*_v_/*F*_m_) analysis. While these have been effective in assessing the threshold for damage, the methods have drawbacks for physiological analyses, especially where laboratory-based biochemical analysis is required (e.g. O_2_ evolution and D1 turnover). In addition, they require disruption of the light treatment, either by destructive sampling or imposition of a sustained dark period. The length of the dark period used for *F*_v_/*F*_m_ measurements as well as the complex nature of the parameter itself cause ambiguity, as described above. The approach required to solve this problem has to be a simple, rapid and non-disruptive method that can test the *in vivo* photoprotective effectiveness of NPQ, regardless of how quickly or slowly it recovers. Here, we describe our recently developed methodology that is aimed at radically changing our understanding of the effectiveness of the NPQ process by quantifying its photoprotective potential in addition to chlorophyll fluorescence induction analysis. The technique will be essential to fully understand the trade-offs between the metabolic cost of photodamage and the reduction in quantum yield caused by engaging NPQ. Theoretical analyses conclude that unbalancing these trade-offs has the potential to substantially reduce plant productivity [15]. In this approach, we use the value of photochemical quenching (*qP*) measured in the dark to monitor the state of active PSII reaction centre, enabling detection of the early signs of photodamage [16,17]. The method allows determination of the amplitude of photoprotective NPQ (pNPQ) and its potential to protect against photodamage. We argue that this approach is more correct than the one that is based only on measurement of the qE component or simply PSII quantum yield. Our analysis allows for accurate quantification of the relationship between the protective component of NPQ and actinic light intensity. This in turn allows estimation of the maximum light intensity tolerated by PSII reaction centres in a plant population, the photoprotective effectiveness of NPQ in plants with different levels of PsbS protein or zeaxanthin and the fraction of captured energy that may be unnecessarily, or ‘wastefully’, dissipated.

## 2. Defining the protective power of non-photochemical chlorophyll fluorescence quenching: photoprotective non-photochemical chlorophyll fluorescence quenching

Pulse amplitude modulated (PAM) fluorescence used in quenching analysis represents a powerful tool for the prompt and detailed study of NPQ and related processes [18,19]. Figure 1 depicts an *Arabidopsis* leaf chlorophyll fluorescence quenching induction measurement. The state of the PSII reaction centres in the dark, the *F*_o_ fluorescence level (all RCIIs are open) and the *F*_m_ level, when all RCIIs are closed by a high-intensity pulse, are indicated in figure 1. The quantum efficiency of PSII can be expressed as:
2.1
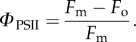
This corresponds to the relative amount of fluorescence that is photochemically quenched due to the activity of the reaction centres. It is important to note that the fluorescence does not immediately return to the initial *F*_o_ level after the pulse. This is due to the fact that the PSII acceptor *Q*_A_ stays reduced for some time. However, it can be promptly oxidized by application of far red light, which excites photosystem I (PSI), causing faster oxidation of the cytochrome *b/f* (Cyt*b*/*f*) complex and the mobile pool of plastoquinones that oxidizes PSII. Following the first pulse of light and a period of dark relaxation, actinic light is applied for about 5 min. Saturating light pulses are used at regular intervals to monitor the level of 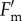. It can be clearly seen (figure 1) that the 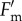 level is being strongly and promptly quenched and reaches a relatively steady state by the end of the illumination period. NPQ is defined as:
2.2
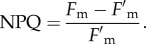


Another parameter that reflects non-photochemical quenching is termed qN and defined as,
2.3
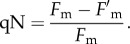
This determines the percentage of quenching in a similar manner to the calculation of *Φ*_PSII_. The NPQ calculation reflects the ratio of the rate constant of NPQ to the sum of the other constants corresponding to all other dissipative pathways, such as fluorescence, internal conversion and interconversion [20]. qE is defined as the energy-dependent, rapidly reversing component of qN or NPQ (figure 1). Normally, this component is considered to recover within 5 min of switching off the actinic light. As shown in figure 1, qE appears to be the major component of NPQ. The remaining portion of NPQ was previously termed qI, or the irreversible NPQ component, to which several processes contribute, as explained above [10–14].

We aimed to develop new methodology capable of distinguishing the extent of photoinhibitory quenching in the qI component and the amplitude of the protective components of NPQ, pNPQ, without using the dark relaxation phase of the quenching analysis [16,17]. In this approach, the extent of photochemical quenching (*qP*) measured in the dark was used to monitor the state of open PSII reaction centres. This enables detection of the early signs of photodamage. It is important to stress again that both NPQ/qE and photodamage to RCIIs diminish the quantum yield of PSII (*Φ*_PSII_) [19,21–23]. First, we addressed the two key aims: (i) the separation of photoprotective and photoinhibitory effects on the PSII yield and (ii) finding out the true value of the NPQ that protects PSII—pNPQ.

*Φ*_PSII_ can be expressed via NPQ using the rate constants of various dissipative processes that determine chlorophyll fluorescence yield levels [19],
2.4
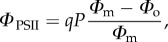
where *Φ*_m_ = *k*_f_/(*k*_f_ + *k*_d_) and *Φ*_o_ = *k*_f_/(*k*_f_ + *k*_d_ + *k*_p_), where *k*_f_, *k*_d_ and *k*_p_ are the rate constants for fluorescence, internal conversion and photochemistry, respectively (for review, see [9]). After a transformation, the following formula is obtained:
2.5
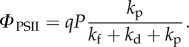


The PSII yield at any point in the dark in the presence of NPQ, therefore, becomes
2.6
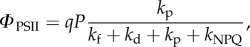
where *k*_NPQ_ is a non-photochemical dissipation rate constant that incorporates the effective quenching rate constant and concentration of the quencher.

Equation (2.6) can be transformed as:
2.7
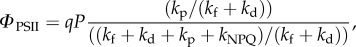
resulting in
2.8
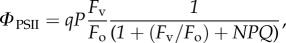
where NPQ = *k*_NPQ_/(*k*_f_ + *k*_d_); *F*_o_ = *k*_p_/(*k*_f_ + *k*_d_ + *k*_p_); and *F*_v_ = *k*_f_ *k*_p_/[(*k*_f_ + *k*_d_)(*k*_f_ + *k*_d_ + *k*_p_)].

Rearranging *F*_v_/*F*_o_ as 1/(*F*_m_/*F*_v_ − 1), the yield in the presence of NPQ in the dark will become
2.9
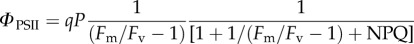
or
2.10
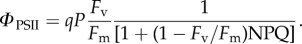


Hence, the PSII quantum yield is expressed as a hyperbolic function of NPQ. In the dark at NPQ = 0, the yield is at its maximum, *Φ*_max_ = *F*_v_/*F*_m_, with all reaction centres open (pre-illumination conditions, *qP* = 1). If NPQ = 2 (in the dark), the yield will decrease from an average of 0.8 to approximately 0.57, while for NPQ = 4, the yield will decrease to approximately 0.44, etc. These considerations will hold only when *qP* = 1 in the dark, i.e. when photodamage is absent. When photodamage takes place, *qP* < 1. This long-term closure of PSII reaction centres undermines the PSII quantum yield and causes its deviation from the theoretical hyperbolic dependency on NPQ.

Photochemical quenching, *qP*, is defined as
2.11
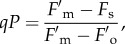
where 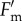, *F*_s_ and 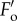 are fluorescence levels measured at maximum, steady-state illumination and dark in the presence of NPQ, respectively. In the dark, immediately after switching off the actinic light and in the presence of far red light, *F*_s_ should in theory become 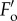, giving *qP* = 1. However, because the damage/closure of RCIIs leads to elevation of 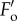, the quenching effect of NPQ is disguised. The calculated 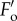 will therefore often become higher than the real 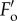. In order to estimate the true 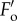 value, the formula of Oxborough & Baker [24] can be applied:
2.12
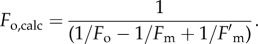


**Figure 2.**
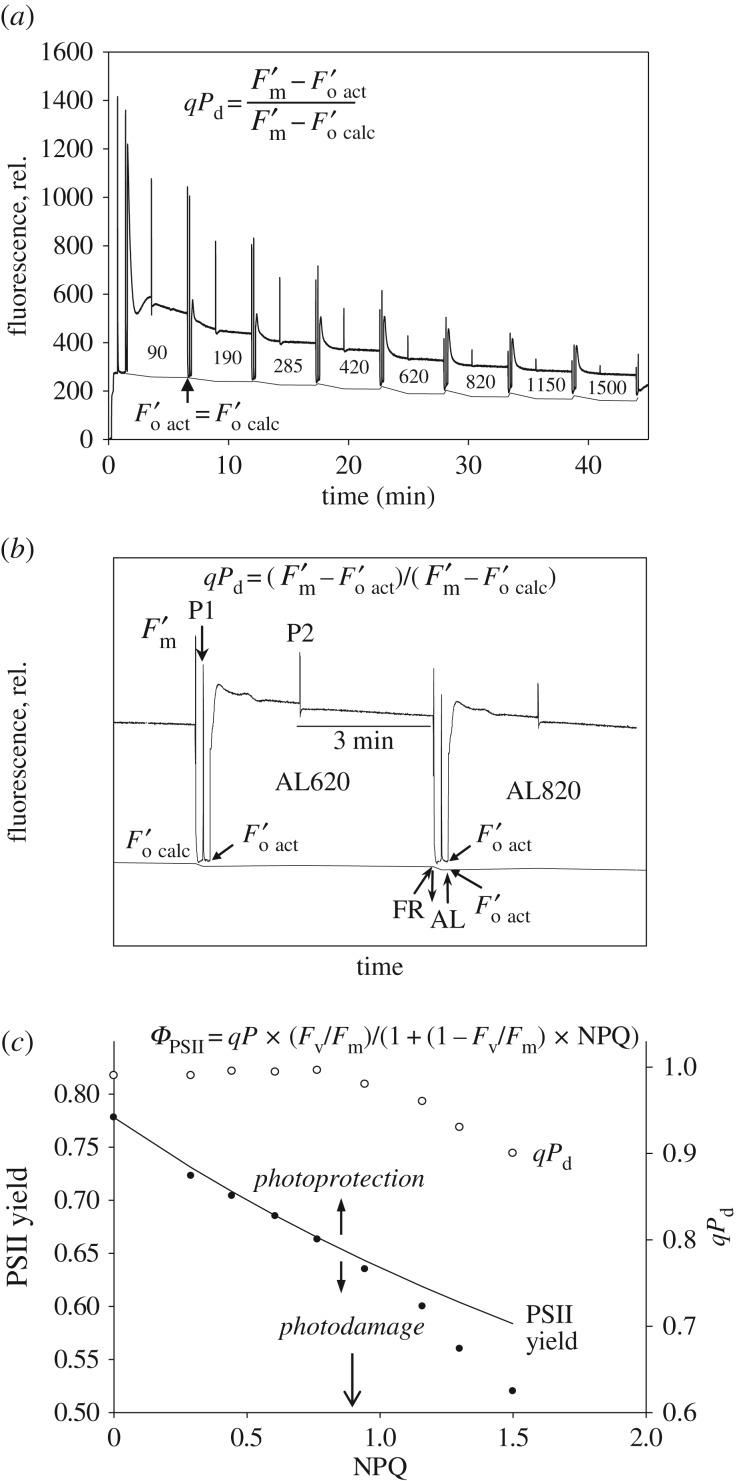
(*a*) Scheme of induction of chlorophyll fluorescence in an *Arabidopsis npq4* mutant plant with an eight-step increasing actinic light (AL) routine. Here, AL intensities of 90, 190, 285, 420, 620, 820, 1150 and 1500 µmol m^–2^ s^–1^ were used. To increase the accuracy of data points, three other intensity ranges were used in other experiments. For detailed explanation of routine development, see refs [17] and [25]. (*b*) A fluorescence induction fragment illustrating the timing and application of AL (upward arrow and downward arrows demonstrate the turning of AL *on* and *off*, respectively), along with saturating pulses (SPs) (P1 and P2). P1 illustrates an SP at the end of the AL cycle in the dark and P2 during AL illumination. FR is far red light illumination applied for 7 s immediately after AL was switched off. The difference between actual and calculated 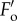 used to calculate *qP*_d_ is also shown. At low AL intensities, 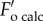 and 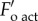 match or are extremely close. Under high light, the two values diverge [17,25]. The timing scheme of the *qP*_d_ calculation and darkness step of the routine was: (AL off) (FR on)–(7 s)–(SP)–(5 s)–(AL on/FR off). (*c*) Relationship between NPQ, PSII actual quantum yield (filled circles) and *qP*_d_ (open circles) taken at the end of each light intensity treatment. The theoretical yield (continuous line) was calculated using formula (2.10).

During photodamage, the value of *qP* in the dark (referred to as *qP*_d_) can be calculated using the measured dark fluorescence (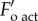) and the true, calculated magnitude, 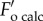, in the following way:
2.13
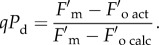
Modern PAM fluorimeters, such as the PAM100 or Junior PAM (Walz), incorporate Oxborough & Baker's formula; therefore, it is easy to monitor both levels of 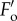 in parallel. Our new quenching procedure is based on the use of a range of gradually increasing actinic light intensities, similar to a light saturation curve procedure, but applied for longer periods of illumination (5 min for each light intensity) with short periods of darkness (10 s) in order to assess *qP*_d_ levels by applying a saturating pulse (figure 2*a*,*b*). At lower actinic light levels, 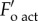 and 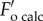 are virtually identical, hence the parameter *qP*_d_ stays close to 1 (figure 1*c*). However, as the light intensity becomes higher, 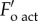 starts to increase above 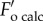 and *qP*_d_ becomes lower than 1. This discrepancy arises from the fact that when RCIIs become closed due to photodamage, they stay closed in the dark, hence they cannot photochemically quench fluorescence, causing an increase in 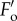, in a similar way to the increase in 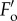 caused by the addition of 3-(3,4-dichlorophenyl)-1,1-dimethylurea (DCMU) or illumination, making this level effectively *F*_s_. Therefore, under these conditions, 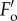 becomes appreciably less quenched in relation to 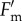, which is manifested in the observed deviation of the experimental from the predicted 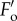 levels, and hence brings the *qP* level down from 1. In parallel, the calculated values for *Φ*_PSII_ (formula (2.5)) at lower actinic light intensities stay very close to the measured yield (figure 2*c*). The measured *Φ*_PSII_ begins to deviate from calculated values at higher light intensities, very close to those that cause the decrease in *qP*_d_ (figure 2*c*, vertical arrows). This deviation and the decrease in *qP*_d_ mark the onset of photodamage and the maximum protective NPQ when all RCIIs still remain intact, pNPQ (down open arrow in figure 2*c*). Higher levels of NPQ, above pNPQ, represent a mixture of pNPQ and qI components. In some rare cases, the level of *qP*_d_ at lower light intensities does not stay constant and becomes gradually greater than 1 [25]. This phenomenon has been thoroughly investigated and it was concluded that it takes place only when part of the LHCII antenna becomes uncoupled from the PSII supercomplex either as a result of the formation of very large antenna sizes (under low light conditions) or on artificial removal of RCIIs in lincomycin-grown plants [25]. In these cases, a correction procedure has been developed and must be used for the accurate determination of *qP*_d_ and pNPQ, respectively [25]. The possibility of interference from the fluorescence of PSI in these measurements, particularly when 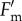 and 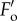 become low (in the case of strong NPQ) [26], has been investigated [27]. It was found that although PSI contributes to *F*_o_ fluorescence, it is also quenched by the NPQ process (as was shown before [13,28]). This prevents immediate *qP*_d_ increase above 1. Taking the above studies into account, we conclude that, because in the vast majority of cases, *qP*_d_ remains constant (approx. 1) across a wide range of non-photoinhibitory light intensities, Oxborough & Baker's formula works very well and there is no interference from PSI fluorescence or antenna detachment from PSII. If the latter takes place, *qP*_d_ becomes higher than 1. Hence, the parameter can also be used as a simple, prompt screen for possible LHCII uncoupling from PSII under certain stress conditions, such as growth at very low light or high temperature stress, which should be further explored.

Critical work has also been undertaken to ensure that the fluorescence parameter *qP*_d_ correlates with the electron transport rates affected by photodamage, as measured by oxygen evolution techniques [29]. This work revealed a linear correlation between the decrease in oxygen evolution rates and *qP*_d_ at photoinhibitory light conditions, and enabled discrimination between the fractions of electron transport affected by downregulation and by photodamage [3]. Therefore, this new, simple procedure based on PAM fluorometry allows quantification of the extent of true photodamage, as well as the protective efficiency of NPQ.

## 3. Photoprotective non-photochemical chlorophyll fluorescence quenching applications so far

### (a) Photoprotection-related qI, the decline in photosystem II quantum yield and photosystem II repair

**Figure 3.**
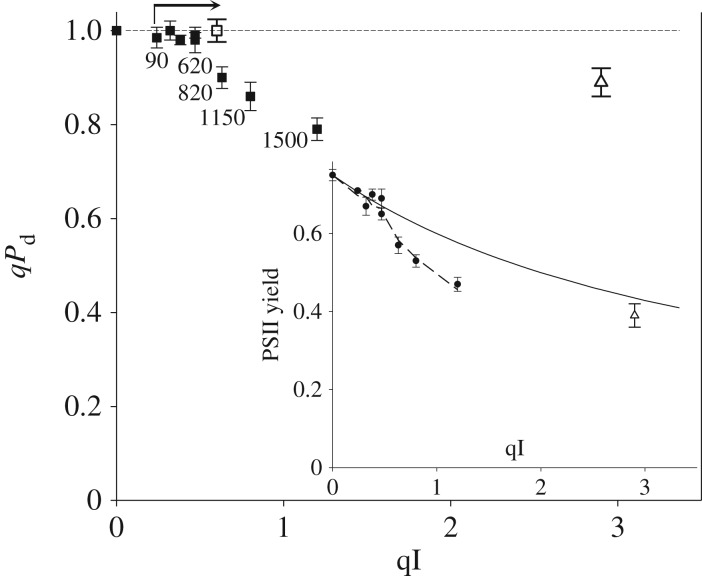
Relationship between *qP*_d_ and qI at the end of the classic fluorescence induction procedure, displayed in figure 1, using a range of actinic light intensities from 90 to 1500 µmol m^–2^ s^–1^ (figure 2*a*, legend). Inset: relationship between PSII quantum yield and qI. The solid line marks the calculated yield using formula (2.5). Open triangles correspond to the *qP*_d_ and PSII quantum yield recorded at the end of 2 h illumination with 1000 µmol m^–2^ s^–1^.

**Figure 4.**
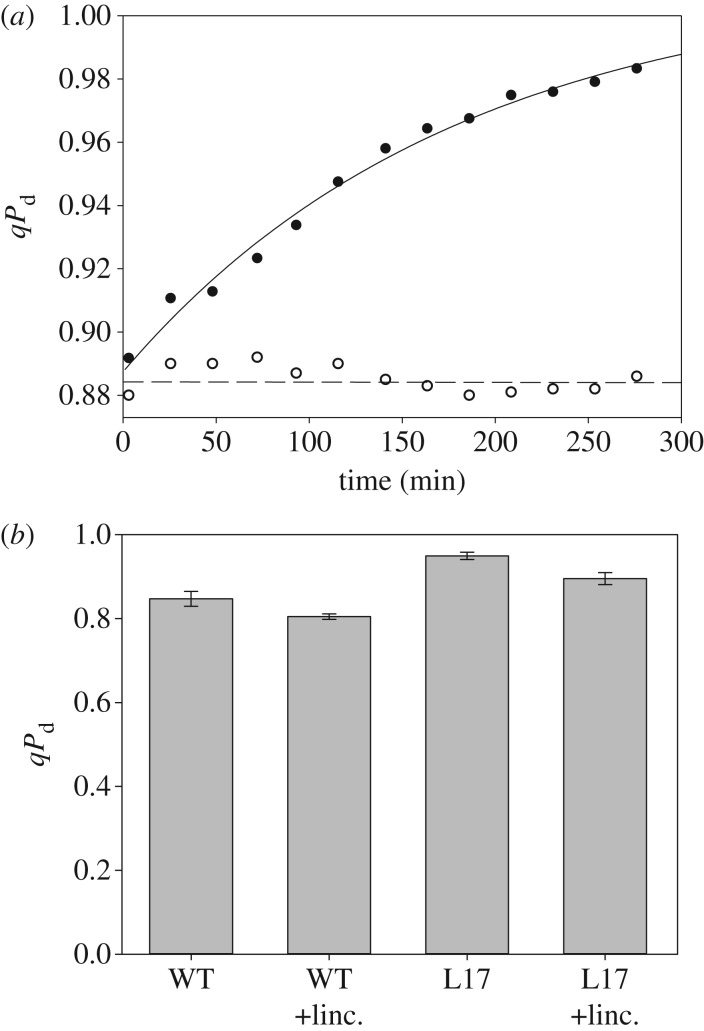
(*a*) The rate of *qP*_d_ recovery after 40 min of 1500 µmol m^–2^ s^–1^ illumination (*Arabidopsis* leaf, WT, dark circles). Light circles correspond to leaf infiltrated with lincomycin before illumination. (*b*) *qP*_d_ levels at the end of the illumination procedure shown in figure 2*a* in wild-type and PsbS overexpressing plants and infiltrated with lincomycin.

First of all, we wanted to distinguish the contribution of true photodamage to RCIIs from the parameter qI and the slowly reversible component of NPQ. For this purpose, we used the standard quenching procedure shown in figure 1, applying actinic light within the range of intensities between 90 and 1500 µmol m^−2^ s^−1^ used in the new pNPQ method (figure 2) and measuring *qP*_d_ and qI at the end of the procedure (figure 1). From these data, the plot of *qP*_d_ versus qI was produced (figure 3). qI was defined in this case as 
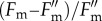
 (figure 1). For the lower light intensities in figure 3, *qP*_d_ was stable at around 1, while qI values reached 0.6. Interestingly, if the duration of treatment was increased to 30 min with a light intensity of 90 µmol m^−2^ s^−1^ (indicated by the vertical line in the bent arrow), qI increased (horizontal line in the bent arrow) up to 0.55 (open square). This qI was found to be of a photoprotective nature (because *qP*_d_ = 1) and it downregulated the PSII quantum yield in a similar fashion to pNPQ, following the relationship predicted by formula (2.10) (see the inset in figure 3). Higher actinic light intensities induced the decline in *qP*_d_ followed by a further increase in qI, which at this stage is composite and contains contributions from true photodamage as well as slowly reversible pNPQ. In many cases, if the treatment with 1500 µmol m^−2^ s^−1^ actinic light lasted for two or more hours, *qP*_d_ started to recover (figure 3, large open triangle), as did the PSII quantum yield (figure 3 inset, open triangle), reaching a value close to the predicted theoretical yield (solid line, figure 3) when photodamage is absent. In fact, the value of qI close to 3 in this case should be a dominant component of sustained protective pNPQ (because the amount of photodamage under these conditions is relatively low, *qP*_d_ approx. 0.9)—revealing its paramount importance in photoprotection. This remarkable trend is likely due to the effect of the D1 recovery system [30], which normally functions on a slow time scale, and pNPQ diminishes the photoinhibitory effect of high light by strongly dissipating a large proportion of its energy. Figure 4*a* represents a proof that the parameter *qP*_d_ recovers fairly slowly in the dark and is associated with the activity of PSII repair, because it is totally lincomycin-sensitive. As far as the establishment of the photodamage is concerned, the process is fairly fast and PSII repair does not significantly contribute to the prevention of the damage onset. Indeed, figure 4*b* represents the values of *qP*_d_ registered after the application of the new quenching procedure (figure 2*a*) on wild-type and the PsbS overexpressing *Arabidopsis* plants (L17). The effect of lincomycin is within 10% only, suggesting that indeed, D1 turnover does not immediately contribute to photoprotection. The repair process is effective over a long time scale of hours (figure 3) even at high light when pNPQ is established and diminishes the damaging effect of this light.

### (b) Minimum photoprotective non-photochemical chlorophyll fluorescence quenching required to protect against a given light intensity

**Figure 5.**
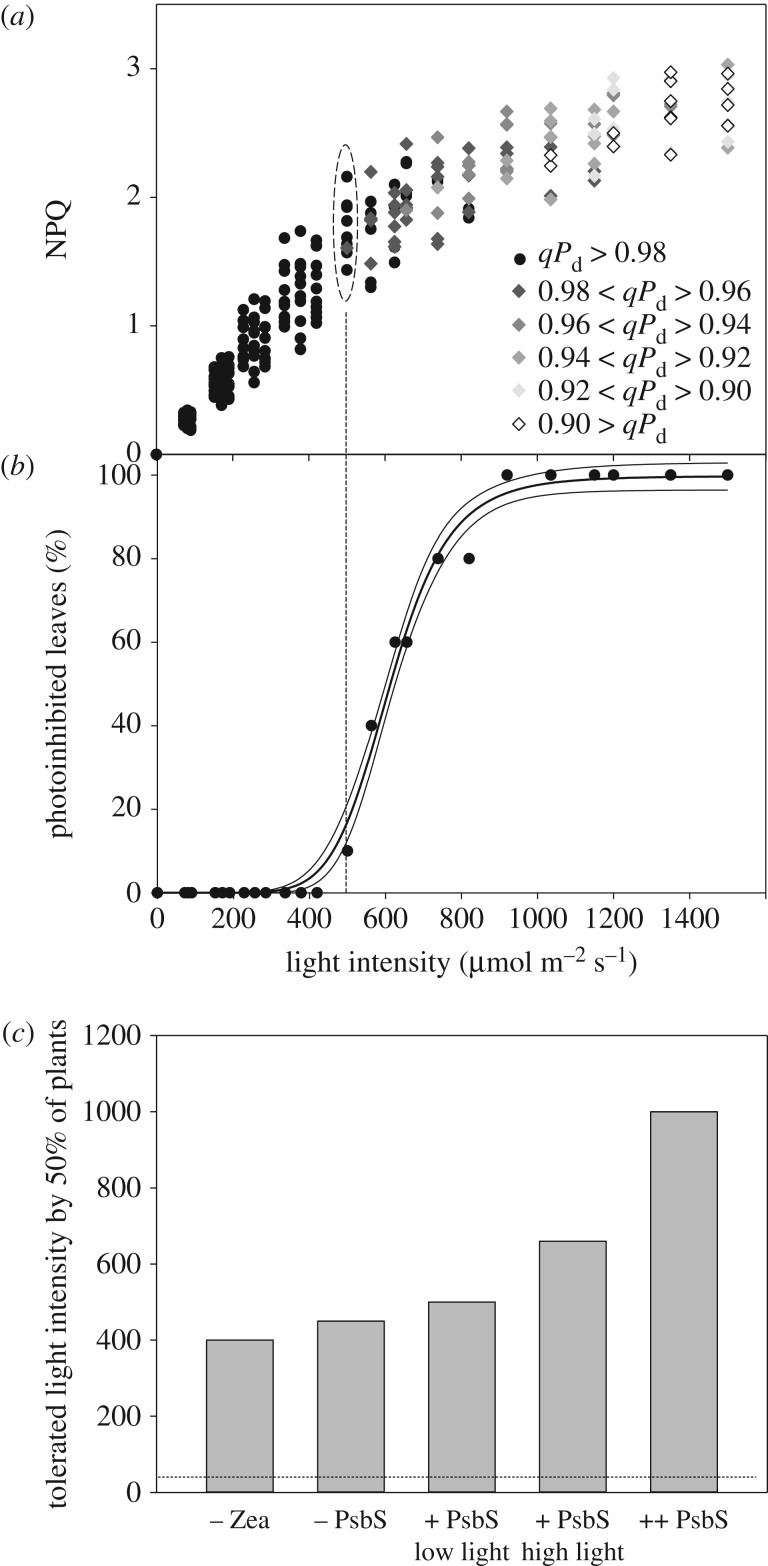
(*a*) The relationship between NPQ, actinic light intensity and *qP*_d_ derived from the measurements using figure 2 experimental scheme on 30 leaves. The legend on the right explains the *qP*_d_ scale of the grey shading of diamond symbols in order to reflect the extent of corresponding photodamage (for other details, see figure 3). (*b*) The relationship between the percentage of leaves affected by photodamage and the actinic light intensity derived from the data shown in figure 5*b*. Solid lines are regression data fit curves with 95% confidence bands obtained using SIGMAPLOT13 software (Systat Software, Chicago, IL, USA). For more details, see [17] and [25]. (*c*) Comparison of light intensity values tolerated by 50% of leaves, obtained using the population light tolerance curves (*b*) for *Arabidopsis* plants lacking zeaxanthin, PsbS protein, grown in shade or high light and overexpressing PsbS protein. The dashed line marks the level of light tolerance in nigericin-infiltrated wild-type leaves.

**Figure 6.**
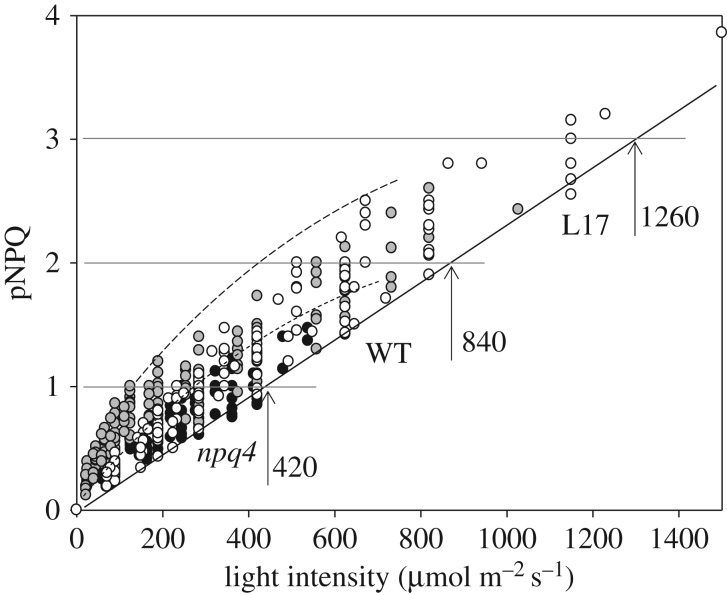
The relationship between the protective component of NPQ (pNPQ) and actinic light intensity taken from figure 5*b* for wild-type (grey), *npq4* (black) and L17 (white) *Arabidopsis* plants. The solid straight line underlines the approximate level of minimum pNPQ required to maintain all PSII reaction centres intact (open in the dark after illumination). Vertical arrows show ‘safe’ light intensity levels, 420, 840 and 1260 µmol m^–2^ s^–1^ for which a minimum NPQ of 1, 2 and 3, respectively, is required to totally protect PSII against photodamage.

The procedure illustrated in figure 2 produced only eight experimental points for NPQ, the PSII quantum yield and *qP*_d_. In order to cover the range of intermediate actinic light intensities and gain reasonable statistics on one plant population, five runs of the procedure were performed on leaves from five different plants and NPQ plotted against light intensity (figure 5*a*) [17,25]. This plot also contains information on *qP*_d_, because NPQ values are depicted by different symbols depending on the class to which the corresponding *qP*_d_ values belonged. NPQ points that correspond to *qP*_d_ values within 1.0–0.98 are depicted by black circles. The NPQ points with corresponding *qP*_d_ values ranging from 0.98 to 0.96 are presented as dark grey diamonds and so on. NPQ values depicted by black circles were defined as pNPQ, because the corresponding *qP*_d_ values were within 2% of 1.0—which was the average experimental value for determination of *qP*. Figure 6 represents a plot of only these pNPQ data as a function of actinic light intensity. The straight line drawn across the lower edge of the data depicts the minimal value of pNPQ required to protect against a given light intensity. For example, in order to have no photodamage (*qP*_d_ within 0.98–1.0) at the light intensity of 420 µmol m^−2^ s^−1^, NPQ should be no lower than 1. This relationship was found to be linear for plants with normal levels of PsbS protein, no PsbS (*npq4*) or plants overexpressing PsbS (L17). Interestingly, although devoid of qE, plants lacking PsbS were able to form pNPQ, in some cases reaching levels of 1.7. Overexpressors of PsbS possessed pNPQ reaching 3.8, which allowed them to tolerate light intensities of 1500 µmol m^−2^ s^−1^, meaning that some *Arabidopsis* plants are potentially capable of tolerating light close to the highest PAR intensity registered on the Earth with no photodamage.

### (c) Population light tolerance curves

In order to obtain information about the light tolerance of the population of leaves/plants in these studies, the data in figure 5*a* can be used to create the population light tolerance plot shown in figure 5*b*. For this purpose, the fraction of all NPQ points showing different extents of photodamage (all diamonds in figure 5*a*) have been divided by the total number of points collected for each light intensity level (encircled by the dashed line, figure 5*a*). This transformation generated one number for each light intensity level applied (marked by the dashed line, figure 5*a*,*b*). For example, for a light intensity of 450 µmol m^−2^ s^−1^, 10 NPQ data points were collected, with only one of them having *qP*_d_ values below the 0.98–1.0 threshold. Hence, 1 divided by 10 gives 10% of samples with signs of photodamage (figure 5*b*). With increasing light intensity from 450 µmol m^−2^ s^−1^, the number of diamonds in the plot of figure 5*a* gradually increased, showing that the population of studied leaves was gradually losing light tolerance, so that eventually none of the studied leaves tolerated 1000 µmol m^−2^ s^−1^ light intensity (figure 5*b*) in the wild-type *Arabidopsis* plants. This procedure was applied to a variety of mutants lacking zeaxanthin or PsbS, overexpressing PsbS or plants grown under different light intensities. Figure 5*c* shows the results of these studies. The light intensity tolerated by 50% of leaves was derived from toleration plots similar to those shown in figure 5*b*. Fifty per cent of plants lacking zeaxanthin tolerated light up to 400 µmol m^−2^ s^−1^, while those lacking PsbS had slightly better 50% light tolerance (450 µmol m^−2^ s^−1^). Removal of NPQ by infiltration with nigericin strongly decreased light tolerance in all types of studied plants to less than 50 µmol m^−2^ s^−1^ (see also [16]), suggesting a key role of NPQ in photoprotection, even in plants lacking zeaxanthin (or both zeaxanthin and lutein [31]) or PsbS protein [32]. Wild-type plants grown under somewhat high light (450 µmol m^−2^ s^−1^) possessed about 40% better tolerance than low-light grown plants (400 µmol m^−2^ s^−1^) (figure 5*c*). Overexpressors of PsbS were the most light tolerant plants. One important conclusion from this line of experiments is that regardless of the NPQ components, zeaxanthin, PsbS or antenna size, etc., the extent of pNPQ relates linearly to the tolerated light intensity.

### (d) Wasteful photoprotective non-photochemical chlorophyll fluorescence quenching?

The plot shown in figure 6 shows that for a given light intensity, pNPQ can vary significantly, particularly for the low intensities, and therefore, sometimes pNPQ can be several times higher than the minimum required for protection. For example, at 200 µmol m^−2^ s^−1^ light, the minimum pNPQ is about 0.5, but pNPQ can also be as high as 1.3 depending on the leaf, meaning that 0.8 pNPQ units correspond to excessive, and potentially wasteful, protection. The question then is why do plants need such excessive protection? There appear to be unknown reasons for this and the significant amount of energy that is not being delivered to RCIIs undermines the yield at low light conditions when every photon counts. Interestingly, plants lacking PsbS form much less wasteful pNPQ (figure 6, black circles). As these plants do not form rapid NPQ, qE, it is possible that qE could be one of the causes for the wasteful quenching. Therefore, for plants growing at low fluctuating light, it is important to optimize their protection, so that the wasteful pNPQ can be kept to a minimum value. Hence, it is important to design future work to assess whether wasteful pNPQ indeed takes place in nature or, most likely, in crops and whether its optimization is a subject to acclimation or genetic engineering. Indeed, as far as crops are concerned, Kromdijk *et al*. [33] report that overexpression of the key modulators of NPQ—PsbS, violaxanthin de-epoxidase and zeaxanthin-epoxidase—result in the creation of tobacco plants that respond much faster to natural light intensity fluctuations. By using the fluorescence approach presented in this review, the authors revealed that the engineered plants waste less light energy in the shade and are also better protected against the damaging effect of bright light compared with control plants [33]. The engineered plants had more efficient photosynthetic electron transport and carbon fixation, greater dry weight and bigger leaf area than control plants. To our knowledge, this was the first direct demonstration that genetically optimizing NPQ could improve crop performance.

### (e) Photoprotective non-photochemical chlorophyll fluorescence quenching in ontogenesis

**Figure 7.**
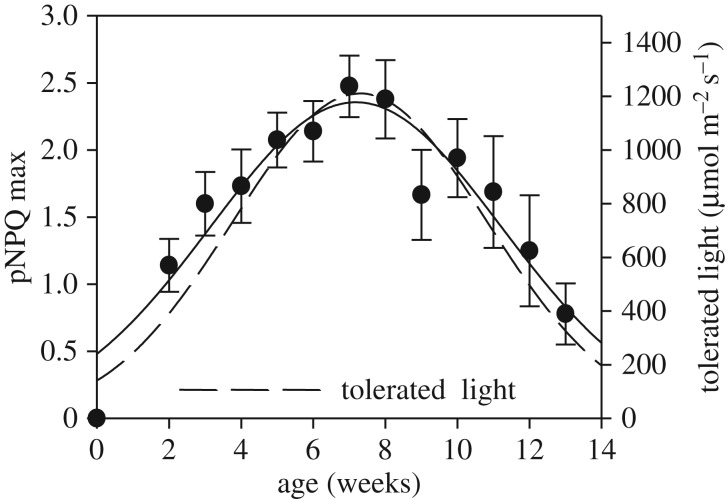
Relationship between maximum pNPQ capacity and plant age. The maximum pNPQ value was considered the first highest non-photochemical fluorescence quenching (NPQ) value that protected 100% of RCIIs, calculated from the relationship between *qP*_d_ and NPQ (figure 1*c*). Error bars show the s.e.m. (*n* = 30). The continuous line is a regression fit curve (peak; Gaussian, three parameter *f* = *a* × exp (−0.5 × ((*x* − *x*_0_)/*b*)^2^)). Dashed lines represent the light intensity tolerated by 50% of leaves.

Recently, we undertook a systematic study of the population light tolerance of *Arabidopsis* plants during ontogenesis [34]. This produced very important quantitative evidence that light tolerance changes during ontogenesis and follows a bell-shaped function with plant age. Figure 7 presents dependencies of maximum pNPQ and light intensity tolerated by 50% of plants depending on plant age. It is important to note that young plants and senescing old plants were most sensitive to light. For old plants, light tolerance was diminished by the fact that the photosynthetic machinery was undergoing a process of degradation, and so NPQ was correspondingly diminished, hence the loss of light tolerance (figure 7). For seedlings, where the photosynthetic machinery was in the process of development, vulnerability to photodamage was a crucial factor and also depended upon pNPQ. Indeed, one- and two-week-old plants were about six and three times less tolerant to light, respectively [34]. Hence, protection of young plants against light stress is another important task for improvement of plant well-being and, potentially, productivity, that should be further explored.

### (f) Imaging photodamage and photoprotection

**Figure 8.**
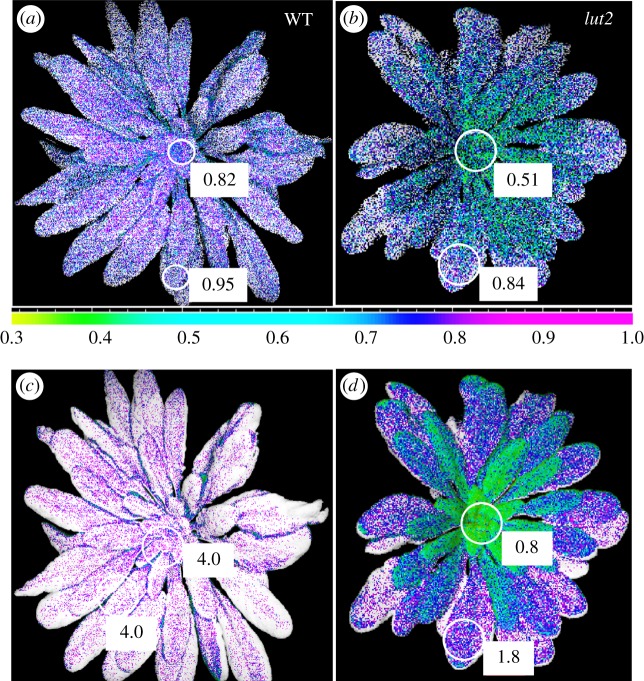
Images of plants depicting *qP*_d_ (*a*,*b*) and NPQ (*c*,*d*) in a false colour scale, obtained using Imaging-PAM (Walz) after the illumination procedure shown in figure 2*a*. (*a,c*) Show wild-type plants and (*b,d*) plants lacking lutein. The colour scale bar applies to all images. However, the maximum amplitude was scaled to 1 for both, *qP*_d_ (coincided with the max. of *qP*_d_ = 1) and NPQ (WT max. NPQ = 4.0 and lut2 = 2.2) images.

Recently, we have adapted the pNPQ procedure for measurements using an Imaging-PAM fluorometer. We have obtained images of plant *qP*_d_ and NPQ values showing the pattern of photodamage and photoprotection in the whole plant. Figure 8*a*,*b* shows images of plant *qP*_d_ levels in false colours recorded following application of the new quenching procedure shown in figure 2 for wild-type *Arabidopsis* and a mutant that lacks lutein (*lut2*). Interestingly, in both cases, the parts of the plant most vulnerable to high light were emerging, young leaves positioned at the centre of the rosette. The mutant suffered the most from high light stress, because its *qP*_d_ was as low as 0.51 in the young and 0.84 in older established leaves, while the wild-type showed corresponding values of 0.82 and 0.95. Better light tolerance in the wild-type plants could be explained by almost double the levels of NPQ in comparison to the mutant (4.0 versus 0.8–1.8). Remarkably, the levels of NPQ in the wild-type were fairly consistent throughout almost all leaves, while for the mutant, NPQ levels in young leaves were less than half that in the older leaves (0.8 versus 1.8). Imaging photodamage and photoprotection appears to be a novel, useful tool for monitoring development of the whole plant canopy at the different stages of ontogenesis, and establishment and degradation of the photosynthetic apparatus.

## 4. Future applications

The aim of this article is to introduce our new methodology, which has been developed to isolate the protective component of NPQ and assess its effectiveness. Using examples, we have shown various types of information that can be obtained using this approach. It conforms to current knowledge about photoprotection and produces data consistent with previous studies on established and characterized types of plant material—high-light versus low-light grown plants, PsbS and zeaxanthin and lutein biosynthesis mutants. Therefore, the validity and merit of this novel approach have now been explored and explained. Going further, the range of future applications seems to be inexhaustible. Potential uses include applying this methodology to various LHC antenna mutants, to wild-type species living in extreme environments, and in combination of high light with temperature, metabolic, water and other stresses. The method can and has to be applied when monitoring crops using the monitoring PAM technology (for equipment that already uses the pNPQ methodology, see: http://www.optisci.com/psp32.html). In this way, we could obtain detailed information about the extent of photodamage and photoprotection and the role of these phenomena in plant development, growth and productivity, eventually attaining the ability to predict the influence of the environment on plant performance and possibly predict the yield of crops. The novel methodology can be used to build up comprehensive light tolerance databases for various current and emerging varieties of crops that are grown outdoors and to optimize growth conditions for crops grown in artificial light environments.

## Data accessibility

This article has no additional data.
